# Non-Negative Matrix Factorization for Learning Alignment-Specific Models of Protein Evolution

**DOI:** 10.1371/journal.pone.0028898

**Published:** 2011-12-22

**Authors:** Ben Murrell, Thomas Weighill, Jan Buys, Robert Ketteringham, Sasha Moola, Gerdus Benade, Lise du Buisson, Daniel Kaliski, Tristan Hands, Konrad Scheffler

**Affiliations:** 1 Biomedical Informatics Research Division, eHealth Research and Innovation Platform, Medical Research Council, Cape Town, Western Cape, South Africa; 2 Stellenbosch University, Stellenbosch, Western Cape, South Africa; 3 University of Cape Town, Cape Town, Western Cape, South Africa; Aarhus University, Denmark

## Abstract

Models of protein evolution currently come in two flavors: generalist and specialist. Generalist models (e.g. PAM, JTT, WAG) adopt a one-size-fits-all approach, where a single model is estimated from a number of different protein alignments. Specialist models (e.g. mtREV, rtREV, HIVbetween) can be estimated when a large quantity of data are available for a single organism or gene, and are intended for use on that organism or gene only. Unsurprisingly, specialist models outperform generalist models, but in most instances there simply are not enough data available to estimate them. We propose a method for estimating alignment-specific models of protein evolution in which the complexity of the model is adapted to suit the richness of the data. Our method uses non-negative matrix factorization (NNMF) to learn a set of basis matrices from a general dataset containing a large number of alignments of different proteins, thus capturing the dimensions of important variation. It then learns a set of weights that are specific to the organism or gene of interest and for which only a smaller dataset is available. Thus the alignment-specific model is obtained as a weighted sum of the basis matrices. Having been constrained to vary along only as many dimensions as the data justify, the model has far fewer parameters than would be required to estimate a specialist model. We show that our NNMF procedure produces models that outperform existing methods on all but one of 50 test alignments. The basis matrices we obtain confirm the expectation that amino acid properties tend to be conserved, and allow us to quantify, on specific alignments, how the strength of conservation varies across different properties. We also apply our new models to phylogeny inference and show that the resulting phylogenies are different from, and have improved likelihood over, those inferred under standard models.

## Introduction

Empirical models of protein evolution, as pioneered by Dayhoff and colleagues [1], [2], have found wide use across varied domains: sequence alignment [3], phylogenetics [4], and as baseline models against which positive selection is detected [5]. These models describe molecular evolution at the amino acid level by quantifying the relative substitution rates between different amino acids. Such rates are an aggregation over multiple distinct phenomena: the structure of the genetic code, which renders some mutations less likely to occur; and differences in the physicochemical properties of the amino acids themselves, which, along with the environment of the organism, will determine which substitutions are deleterious, tolerated or adaptive.

The original approach by Dayhoff *et al.* used a maximum parsimony procedure to reconstruct the ancestral sequences and phylogeny for a collection of protein families and counted the amino acid substitutions across this phylogeny. Their PAM (point accepted mutation) matrices were derived from rates of amino acid exchange estimated from these counts. Jones et al. [6] automated a similar procedure which ran on a much larger dataset, producing the JTT amino acid rate matrix. A further refinement to these “counting” methods was contributed by Kosiol and Goldman [7]. Whelan and Goldman [8] made use of a maximum likelihood approach which, unlike the counting methods mentioned above, finds the amino acid substitution matrix while simultaneously optimizing the branch lengths of the phylogeny, thus incorporating the possibility of multiple substitutions taking place along any given branch. In constructing their WAG matrix, they applied an approximation of this technique to a large dataset.

The above models are generalist in that they use the same set of relative amino acid exchangeabilities for all genes and all organisms. However, since these exchangeabilities can vary considerably between genes and/or organisms, researchers have also constructed specialist models. Such models are estimated from – and intended for use on – a specific gene, organism or genetic code. Adachi and Hasegawa [9] estimated an empirical amino acid substitution rate matrix for mitochondrial DNA-encoded proteins, using the maximum likelihood method on a dataset consisting of mtDNA-encoded sequences from vertebrate species. Yang et al. [10] used a similar technique to derive a substitution rate matrix from the mtDNA mammalian dataset of Cao et al. [11]. Both of these are intended for use only on mitochondrial sequences. Dimmic et al. [12] optimized an amino acid substitution rate matrix via maximum likelihood, using a set of retroviral pol protein sequences. Nickle et al. [13] derived two substitution rate matrices with maximum likelihood, each using different HIV protein sequence datasets. The first matrix (HIVwithin) was derived by applying maximum likelihood to pairs of within-individual protein sequences, while the second (HIVbetween) made use of a set of consensus sequences obtained from a population of individuals. In all cases, specialist models fit alignments for their particular system better than generalist models.

Specialist models are better than generalist ones, but specialist models simply don't exist for most alignments. If the alignment is very large, one can estimate a fully parameterized general reversible model (often referred to as REV), which involves estimating 190 parameters. With most alignments, however, this will be severely over-parameterized. Computational biologists who want to analyze a single alignment for which a specialist model has not been constructed are therefore forced to resort to using a generalist model. This is the problem we seek to address: constructing alignment-specific models of protein evolution without over-fitting, allowing the model to be just as complex as the data justify.

We investigate a compromise between generalist and specialist models by first extracting, from a large dataset, the important dimensions of variation in amino acid substitution rates, and then using these to constrain our models. We propose the following three step approach: First, we estimate a separate REV amino acid rate matrix for each of a number of reasonably large alignments. These provide a library of specialist models, each with 190 rate parameters. Second, we apply non-negative matrix factorization – a dimensionality reduction technique – to find a smaller set of ‘basis’ rate matrices, whose non-negative weighted combinations best approximate the original REV estimates. Finally, for a new alignment (which is not contained in the original dataset and may be relatively small), we model the amino acid rate matrix as a weighted combination of our set of basis matrices. During this final step, we optimize over both the number of combination weights and their values. NNMF is thus used to approximate the space of useful models, reducing the number of parameters required to explore it. Rate matrices for specific alignments are estimated by searching within this lower-dimensional parameter space.

The basis matrices obtained by our NNMF procedure are interesting in that they reveal a set of components from which the eventual rate matrices are comprised – each alignment-specific rate matrix is the sum of positive multiples of the basis matrices. By measuring, for each basis matrix, the correlation between the amino acid exchangeabilities and the strength of the different physicochemical properties of the amino acids being exchanged, we obtain an indication of how the degree of conservation of the different properties varies between different alignments.

Using a separate test dataset, we show that models estimated through our procedure outperform existing models in terms of Akaike's information criterion (AIC) on all but one of 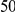 alignments tested. Finally, we use our models to infer phylogenies and show that this leads to phylogenetic trees that are structurally different and have higher likelihood than maximum likelihood trees obtained using standard methods.

## Methods

We start by briefly reviewing phylogenetic models of protein evolution. Substitutions along every branch of a phylogenetic tree are described by a continuous time Markov process, defined by an instantaneous rate matrix, 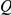. The elements 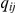 are the rates of substituting amino acid 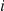 with amino acid 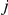. From the rate matrix 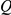 and the length of a branch in the phylogeny, 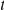, a transition probability matrix for that branch can be calculated using the matrix exponential:
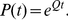
(1)


The constraint 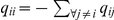 is required for 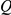 to be a valid Markov process generator. The 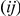 elements of 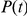 describe the probabilities of substituting amino acid 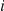 with amino acid 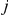 after time 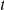. With these transition probabilities along the branches of a phylogeny, the likelihood of an alignment can be calculated using Felsenstein's pruning algorithm [4].

We assume the Markov process is reversible: that is, 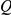 can be decomposed into the product of a symmetric matrix 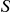 and a diagonal matrix 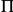, where the elements of the diagonal of 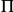, 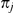, are the equilibrium frequencies for the 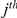 amino acid in the Markov process defined by 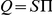, with 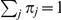. Throughout this paper we adopt a common approximation by estimating the equilibrium frequencies 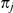 as the empirical amino acid frequencies counted across all sites in the alignment.


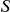
 is the 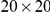 symmetric amino acid exchangeability matrix. Given the symmetry and the constraints on the diagonal elements, this leaves 190 parameters that need to be specified to define the model of protein evolution over a given phylogeny. Our focus in this study is the estimation of these parameters.

### Estimating reversible protein models

To characterize the important dimensions of relative substitution rate variation, we first estimate a general reversible (REV) model – where the 190 parameters of 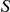 are estimated by maximum likelihood – from each of a large number 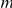 of large alignments. We use the procedure described in [13] to estimate a REV model for each alignment. For computational reasons we use a single rate class, ignoring site-to-site amino acid rate variation (although we show that this can be added at a later stage of our procedure).

### Non-negative matrix factorization

**Figure 1:**
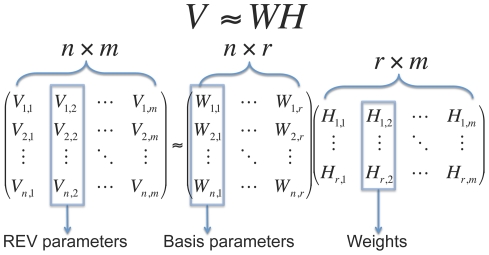
Non-negative matrix factorization.

Non-negative matrix factorization (NNMF) is a tool for dimensionality reduction [14], [15] of datasets in which the values, like the rates in the rate matrix 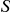, are constrained to be non-negative. Instead of applying it to data, we use it to reduce the dimensionality of our models. We start by arranging the parameters of each specialist REV model into a vector of dimension 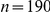. The set of 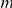 such vectors combine to form a 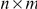 matrix 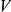 (Figure 1, Table 1) representing the full set of specialist rate matrices. For a given factorization rank 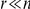, the NNMF procedure now finds an 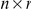 matrix 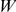 and an 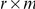 matrix 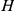 such that 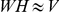. This is done by minimizing an objective function: we chose to minimize the sum of squared differences between 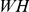 and 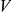.

**Table 1:** Interpretation of the matrix factorization in Figure 1.

m	Number of training alignments
n	Number of parameters per rate matrix (190)
r	Number of basis matrices
Column of V	Specialist REV model corresponding to one training alignment
V	Library of specialist REV models
Column of W	One basis matrix
W	Set of 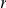 basis matrices
Column of H	Set of weights with which to combine basis matrices to obtain model for one training alignment
H	Set of weights for training dataset


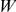
 now represents a set of 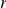 basis matrices: each column contains the 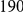 parameters of a single basis matrix, and the 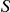 matrix for any of the training alignments can be reconstructed (approximately) by forming a weighted sum over these basis matrices. The weights in this sum are stored in the column of 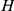 corresponding to the training alignment in question. One way of interpreting the factorization is that the set of basis matrices in 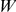 captures the dimensions of important variation between different rate matrices representing the training alignments, so that they form a set of components out of which any of the rate matrices can be built up. Our key assumption is that this will also be the case for alignments not in the training dataset: after paying the fixed cost of learning the 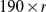 parameters in 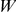 from the training dataset, we propose to represent any alignment using only 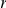 weight parameters instead of 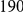 independent rate parameters (Figure 2).

**Figure 2:**
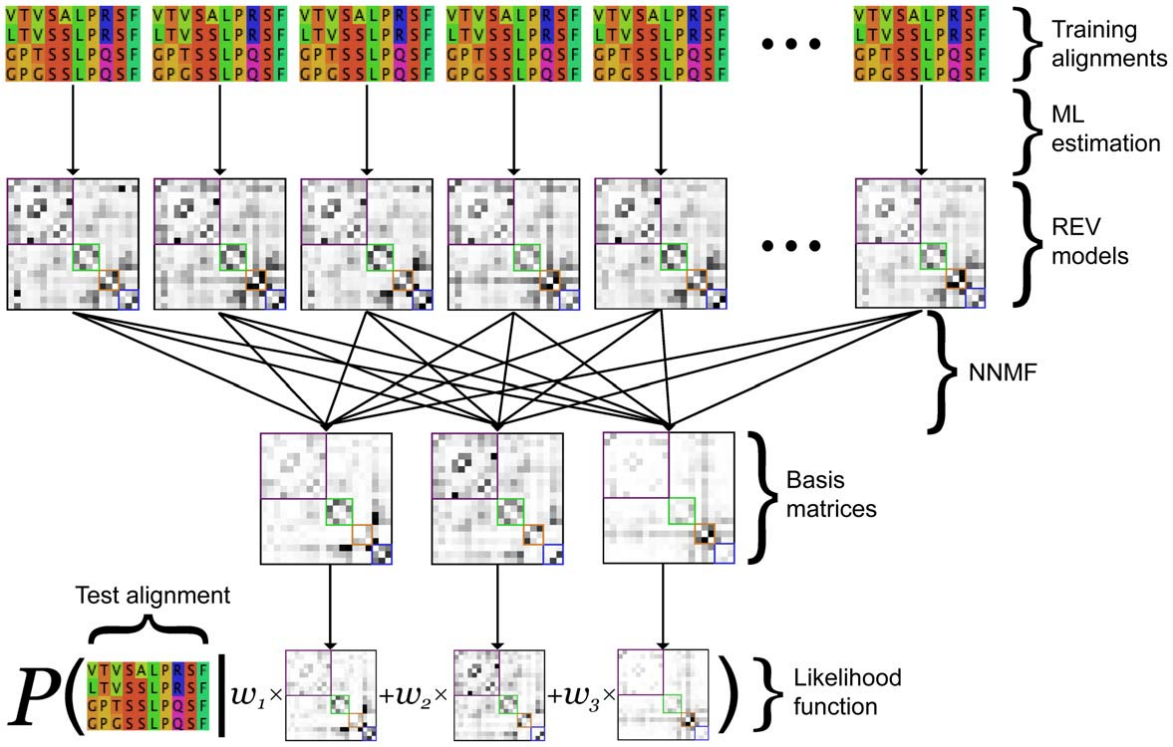
Learning models of protein evolution with NNMF. A schematic overview of the procedure.

NNMF proceeds by an iterative algorithm, converging on a local minimum of the sum of squared error. It is thus potentially sensitive to initial conditions. To ensure decent performance, we began with 20 different random initial conditions and optimized the factorization for 2000 iterations each. The best resulting factorization was then further refined for an additional 5000 iterations.

### Fitting basis models to new data: optimizing over combination weights

Given a collection of 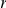 basis exchangeability matrices, 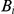 (the columns of 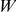 arranged as a reversible rate matrix), their associated weights, 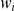, where i goes from 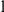 to 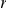, a combined exchangeability matrix 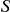 is parameterized by:
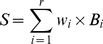
(2)


We add the constraint that 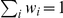: since rate and time are confounded, and since the branch lengths are free parameters, this does not entail loss of generality. With a new test alignment (that was not included in the original factorization over the training data) and a collection of basis rate matrices, we can now optimize the weights 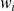 (and branch lengths) to obtain the maximum likelihood combined model for the alignment. This is in contrast to model selection approaches such as ProtTest [16] which select a single model from a collection of existing models. Importantly, the combined model can itself be represented as a single numeric rate matrix, and can thus be used by any application that allows for custom amino acid rate matrices, such as HyPhy [17], PAML [18] or PhyML [19].

The flagship method presented in this paper applies this approach to our NNMF-estimated basis matrices (we refer to this method as “NNMF”). We also introduce a method that uses the same mixture approach, but differs from NNMF, in that it uses a collection of existing numeric rate matrices for its basis matrices , and we name the resulting model the ‘Mixture of Existing Rates’ (MOER) model. For any given test alignment, both models use mixture components that are fixed in advance, but NNMF obtains these by factorizing a large dataset, while MOER uses existing “average” model estimates. The models we chose to combine in MOER are those available by default in the HyPhy software package: Dayhoff, JTT, WAG, rtREV, mtMAM, mtREV, HIVwithin and HIVbetween. For both NNMF and MOER, the equilibrium frequencies used when modeling the test alignments are estimated from the amino acid counts.

These are also the fixed rate models we use as a comparison for NNMF and MOER to asses the performance of our methods, since they are standardly used in the literature. Under a fixed rate model, the branch lengths are optimized to maximize the likelihood, but the exchangeability matrix itself has no flexibility. Each fixed rate model is a special case of MOER, when the weights for all but a single matrix go to 0. MOER will thus always obtain better likelihoods than any single fixed-rate model, but our model comparison measure will penalize against the extra parameters if they prove unnecessary.

### Selecting the optimal factorization rank for a given alignment

The NNMF decomposition requires the specification of a factorization rank: the number of basis matrices to be estimated. Since the optimal number of basis matrices for a new alignment depends on the details of that alignment – larger alignments can justify more parameters – no single factorization will suffice. Instead, we obtain factorizations for a range of different ranks. To select the best NNMF model for each new alignment, we maximize the likelihood function for every rank, and select the model with the best (minimum) AICc(Akaike's information criterion with a small sample correction [20]) score, which prevents over-fitting by penalizing the inclusion of additional parameters:
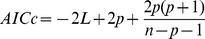
(3)where 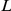 is the log-likelihood, 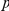 is the number of parameters and 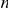 is the number of observations. Counting the number of observations is not straightforward: taking the total number of characters in the alignment is problematic because amino acids at the same site are extremely correlated. (If one were to do this, one could add duplicate sequences which would increase the number of observations without being at all informative.) Instead, we use the number of sites as the number of observations. This can lead to problems when branch lengths are included as parameters, because as the number of branches approaches the number of sites (specifically, when 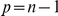), the second order term becomes undefined. This is not just a theoretical concern: it actually occurs for one of our test alignments. To remedy this, we exclude branch lengths from our model parameter count. Excluding branch lengths as parameters when extra taxa are not counted as extra observations makes intuitive sense: adding taxa increases the number of branch length parameters to be estimated while providing the required information to estimate those parameters, but is not correspondingly informative for estimation of the other model parameters. For further discussion of these issues, see [21].

### Phylogeny comparison

To determine whether improvements in model fit would make a difference to the topology of the inferred phylogeny, we compared the best NNMF model to WAG, the existing amino acid model with the best overall fit on our 50 test alignments. We constructed 50 phylogenies using WAG, and 50 using the best NNMF model. Topology search was performed in PhyML [19] with nearest-neighbor interchange plus subtree pruning and regrafting, and we disallowed rate variation due to computational restrictions. We compared topologies under the Robinson-Foulds symmetric difference [22] using PHYLIP [23].

### Data

**Figure 3:**
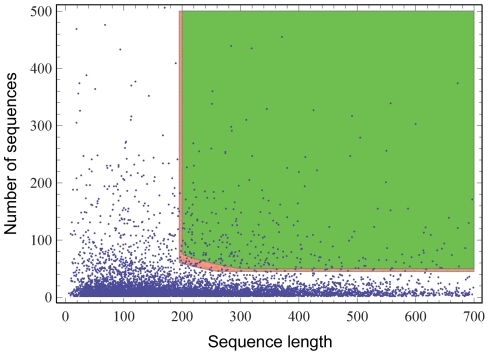
Selecting the larger Pandit alignments. Each blue dot represents an alignment in the Pandit database. The green region covers the alignments used in the training set, and the thin red region covers those in the test set.

Training and test alignments were selected from the Pandit database [24], with the selection based on the size of the alignments (Figure 3). For our training dataset (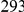 alignments in total) we used all alignments with number of sequences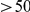, alignment length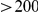 and number of sequences
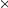
alignment length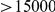, with the exception of one very large alignment (number of sequences
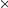
alignment length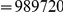) that exceeded our computational resources. The number of sequences per alignment ranged from 51 to 797, with a median of 95 and an inter-quartile range (IQR) of 77. The alignment length ranged from 201 to 1767, with a median of 339 and an IQR of 230.75. All trees used to train the models were also obtained from the Pandit database.

We then adjusted our size criteria to yield a test dataset containing the 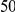 “next largest” alignments: number of sequences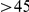, alignment length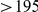, number of sequences
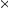
alignment length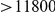, but excluding all training alignments. The number of sequences per alignment ranged from 46 to 182, with a median of 51 and an IQR of 12. The alignment length ranged from 196 to 926, with a median of 249 and an IQR of 207. Trees were again obtained from the Pandit database.

### Implementation

HyPhy [17] was used to estimating the original 293 REV models from the Pandit alignments, using code from [13]. The non-negative matrix factorization was performed in Matlab. Optimizing over basis matrix combination weights for all factorization ranks was performed in HyPhy, as was the comparison of protein models. HyPhy Batch Language (HBL) code for optimizing over combination weights is available online (www.cs.sun.ac.za/~bmurrell/nnmf/), along with the basis matrices. A web script for converting from this output to a rate matrix that is usable by PAML and PhyML is also available at the same url.

## Results

### The basis matrices

**Figure 4:**
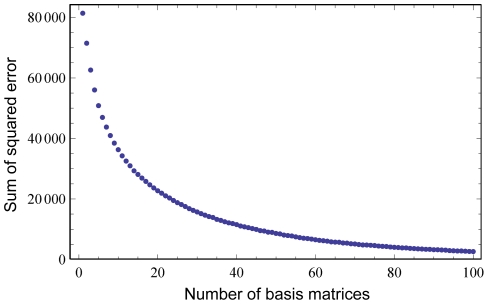
Convergence of NNMF. The sum of squared error decreases as more basis matrices are included.

**Figure 5:**
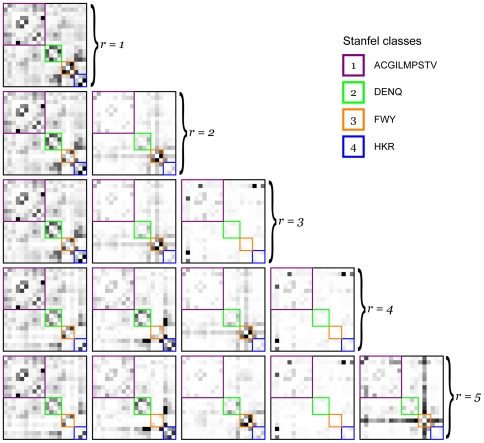
NNMF basis matrices. The set of NNMF basis matrices obtained for ranks ranging from 1 to 5. Amino acids are ordered according to their Stanfel classification [25]. Rates are indicated in grayscale, with pure white being a rate of zero and pure black being the maximum rate in the matrix.

We first consider the set of basis matrices obtained on the training alignments. Figure 4 shows that, as expected, the sum of squared errors decreases as the number of basis matrices increases. To investigate the first few sets of basis matrices, we use the Stanfel classification [25] of amino acids according to their physicochemical properties. Figure 5 shows the basis matrices obtained for the first 5 ranks, with the amino acid ordering chosen so as to group amino acids with similar properties together. We observe that, when one or two rate classes are used, the larger rates (darker squares) occur more frequently within the same class than between classes. Thus these rate matrices capture the fact that, on average, physicochemical properties tend to be conserved.

As more rate matrices are added, the variation between different alignments becomes better resolved. By the third factorization (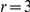), a basis matrix occurs with larger rates (involving Cysteine) occurring between classes. This reflects that, in some alignments, these rates are accelerated while in other alignments they are not: the NNMF analysis indicates that whether these rates are high or low is an important dimension of variation across the training alignments. We also notice that the exchangeabilities of Cysteine with other amino acids are not elevated independently: in alignments where the Cysteine
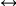
Histidine exchangeability is elevated, the Cysteine
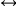
Leucine and Cysteine
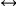
Arginine exchangeabilities also tend to be elevated. This may reflect that the properties under conservation in these alignments, along with the relative importances of these properties, differ from those used to define the Stanfel classification; rather than speculating about the underlying biochemistry, we restrict ourselves to pointing out that the set of basis matrices provides a far richer description of amino acid exchangeability, and how this varies between alignments, than can be achieved by classifying the amino acids into a predefined set of non-overlapping categories.

With 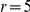 we see that Tryptophan has increased exchangeability with most other amino acids in a subset of alignments. It would be interesting to establish the underlying causes of such effects; for now we merely note that they are easily observable. Inspection of the basis matrices for larger values of 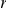 would lead to many similar observations.

**Figure 6:**
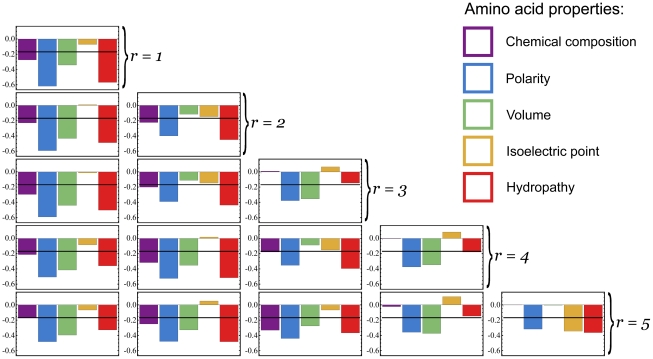
NNMF basis matrices correlate with amino acid properties. The correlations between amino acid properties and the basis matrices. The horizontal black line (at −0.16867) indicates the threshold for significant negative correlation (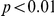, one tailed, 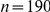).

Figure 6 displays the correlations of the rates in the basis matrices for the first 5 factorizations with 5 amino acid properties (chemical composition, polarity, volume, isoelectric point and hydropathy). The values for these properties were obtained from [26]. Here we are correlating the rate of substitution between two amino acids with the difference between their values of the relevant property. As expected, negative correlations predominate: amino acids with larger differences are less frequently exchanged. The horizontal black line (at −0.169) indicates the threshold for significant negative correlation (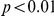, one-tailed correlation test, 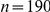). The relationships between the chemical properties and the basis matrices clearly vary across the factorizations. For instance, the fifth basis matrix for 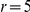 (which as we saw corresponds to an elevation of the overall exchangeability of Tryptophan) corresponds with significant conservation of polarity, isoelectric point and hydropathy (evidently, exchanging Tryptophan for another amino acid does not affect these properties very much on average), but no conservation of chemical composition or volume (Tryptophan substitutions do affect these properties).

### NNMF consistently yields better models than other approaches

**Figure 7:**
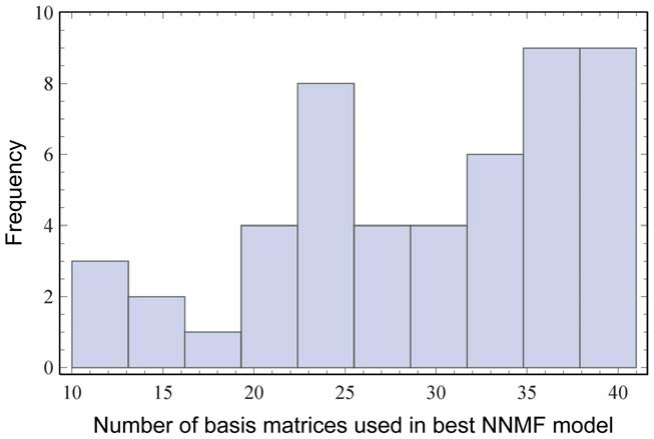
Distribution of the optimal number of basis matrices. The number of basis matrices that minimized the AICc across 50 test alignments.

For each of the 50 Pandit test alignments, we optimized the weight vectors and computed the AICc scores for the first 40 factorizations (from 1 to 40 basis matrices; we stopped at 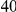 because finding weights by maximum likelihood is computationally intensive, taking, for example, 2 to 3 hours to get up to 40 with datasets of around 600 codons and 50 sequences, but taking substantially longer as larger numbers of basis matrices are considered). The number of basis matrices that minimized the AICc was dependent on the alignment. This optimal number ranged from 11 to 40, with a median of 30.5 and an interquartile range (IQR) of 11. Figure 7 shows the distribution of the optimal number of basis matrices for the best NNMF model across all 50 test datasets.

From the 50 test datasets, we also computed AICc scores for the MOER model, as well as for each named amino acid model implemented in HyPhy, the REV model and the REV 1-step model (which fixes to 0 the rates of all amino acid substitutions that require more than one nucleotide change). Following Burnham and Anderson [27], we compute 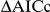 scores, which are the AICc scores for each model minus the best AICc for that dataset. The best model will thus have 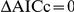. Models with 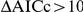 have “essentially no support” [27]. Table 2 summarizes the frequency of each model's 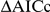 scores. The NNMF procedure for finding models appears to consistently outperform the others, obtaining the best AICc on 49 of 50 datasets. REV won on a single alignment, which, unsurprisingly, was the largest alignment and thus able to justify the full 190 rate parameters. The best NNMF model on this dataset had a 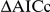 of 0.34, which indicates that it has only slightly less support than REV.

**Table 2:** 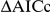
 scores for all models.

	0	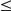 2	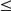 4	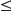 8	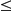 16	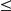 32	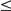 64	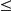 128	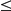 256	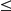 512	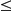 1024	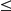 2048	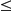 4096	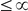
NNMF	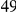	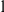												
MOER							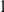	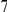	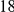	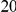	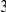	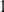		
REV	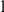							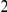	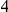	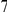	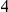	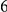	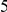	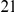
REV-1 step									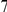	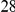	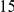			
Equal Input												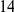	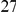	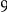
Dayhoff										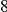	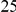	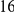	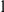	
JTT									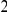	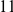	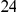	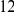	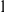	
WAG								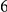	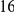	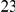	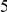			
rtREV									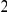	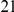	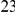	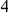		
mtMAM												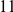	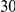	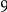
mtREV 24											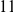	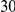	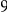	
HIVwithin												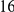	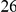	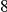
HIVbetween											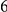	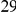	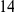	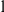

Each table entry is the number of datasets with 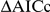 in that range. For any dataset, the best model has 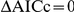. A model with 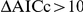 has essentially no support.

Our approach of selecting the factorization rank using AICc is equivalent to selecting the best of the 40 NNMF models under consideration. Such a model selection step arguably gives NNMF an unfair advantage over the other models; although it is not standard procedure in the AIC literature, it may be more correct to add a penalty to the AICc scores of NNMF. Though not strictly appropriate for this context, a Bayesian argument can be used to estimate the appropriate size of this penalty: if we are comparing NNMF as a whole procedure against a single other model and we distribute the prior probability for NNMF uniformly over the 40 NNMF candidate models, we would introduce a penalty of at most 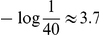 to the resulting marginal likelihood for the NNMF procedure. This would amount to a maximum AICc penalty of approximately 7.4 to the scores for NNMF. Applying this penalty in Table 2 does not substantially affect the results. Furthermore, if we fix the number of basis matrices used (we picked 20) for all alignments, we still outperform WAG (the best overall fixed model) on all alignments with a median AICc improvement of 225 points. This is despite removing the model's ability to adapt its complexity to suit the data. That the improvement remains is not surprising: even a fixed amount of flexibility is better than none, as long as it does not require too many parameters for any particular alignment.

It is also interesting to look at the AICc scores excluding the NNMF models (Table 3). Here we see MOER finding the best model most often (21/50 times), with WAG a close second (15/50) and REV and REV 1-step next with 8/50 and 6/50 respectively. Predictably, most of the specialist models (mtMAM, mtREV 24, HIVwithin and HIVbetween) perform badly on datasets they were not intended for, with the exception of rtREV, which outperforms both JTT and Dayhoff (38, 10 and 2 wins respectively). Interestingly, in [13], rtREV was outperformed by generalist models WAG and JTT on HIV alignments containing the reverse transcriptase protein.

**Table 3:** 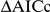
 scores without NNMF.

	0	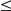 2	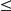 4	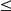 8	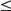 16	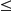 32	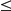 64	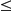 128	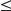 256	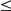 512	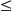 1024	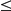 2048	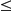 4096	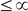
MOER	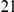			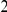	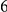	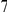	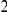	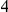	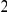	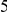		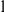		
REV	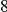						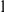	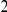	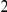	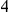	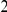	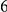	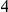	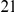
REV-1 step	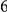					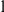	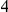	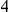	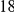	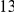	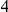			
Equal Input												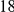	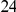	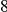
Dayhoff								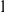	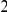	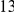	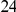	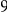	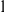	
JTT								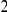	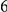	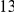	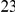	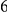		
WAG	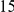	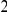	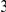	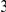	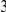	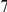	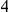	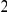	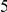	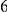				
rtREV			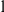					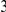	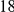	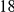	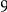	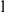		
mtMAM												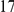	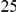	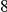
mtREV 24											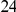	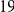	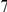	
HIVwithin											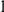	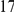	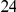	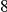
HIVbetween											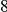	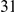	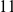	

Each table entry is the number of datasets with 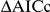 in that range. For any dataset, the best model has 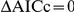. A model with 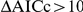 has essentially no support.

The use of constant rates across sites is an unrealistic assumption. It is possible to incorporate rate variation in a Random Effects Likelihood (REL) framework, where the rate at a site is modeled as a random draw from a discretized distribution. This incurs additional computational expense proportional to the number of rate categories used. To demonstrate that our results hold when rate variation is incorporated into all models, we randomly selected 10 test alignments and accounted for rate variation using a discretized gamma distribution with 4 rate categories. Table 4 displays the results for these 10 datasets. The conclusions are unchanged, and NNMF yields the best models for all 10 alignments.

**Table 4:** 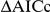
 for all models with gamma rate variation (4 categories).

	0	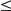 2	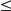 4	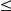 8	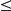 16	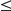 32	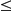 64	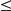 128	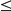 256	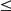 512	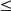 1024	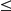 2048	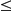 4096	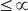
NNMF	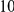													
MOER						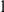	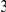	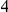	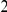					
REV										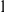		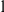		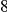
REV-1 step										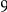	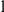			
Equal Input												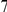	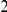	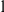
Dayhoff									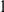	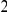	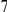			
JTT									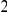	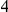	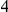			
WAG							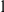	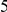	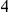					
rtREV								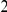	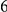	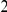				
mtMAM												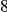	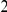	
mtREV 24											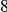	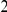		
HIVwithin												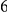	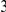	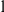
HIVbetween											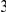	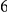	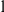	

Each table entry is the number of datasets with 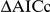 in that range. For any dataset, the best model has 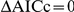. A model with 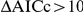 has essentially no support.

### NNMF models yield different phylogenies with better likelihoods

The Robinson-Foulds distance between the trees found using the WAG matrix and those found using the best NNMF model ranged from 0 to 98, with a median of 19 and an IQR of 24. This shows that the choice of model makes a difference to the estimated phylogeny. The NMMF phylogenies also have much higher likelihoods (and lower AICc scores) than the phylogenies estimated using WAG. When using maximum likelihood as a criterion for optimizing phylogenies, topologies and models that yield higher likelihoods should be preferred. This is not direct evidence that the NNMF procedure leads to more accurate trees (which would be difficult to demonstrate for a convincingly large sample), but it does suggest that we should expect such an improvement.

**Figure 8:**
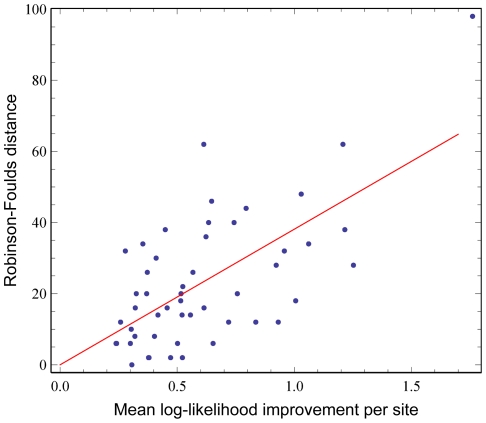
Likelihood improvement predicts phylogenetic difference. The difference between phylogenies increases as the mean likelihood difference per site between NNMF and WAG increases. 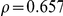, (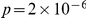, randomization test with 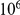 replicates). Assuming intercept of 0, slope = 38.1. Without this assumption, intercept = −0.31, slope = 38.5.

Bigger differences in likelihoods predict bigger differences in phylogenies. Figure 8 shows the relationship between the mean log-likelihood improvement per site for a given alignment and the Robinson-Foulds distance between the two resulting topologies. There is a strong positive correlation with 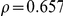, 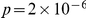 (randomization test with 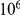 replicates). The slope of the best fitting line is 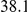, indicating a Robinson-Foulds distance increase of 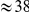 for each log-likelihood per-site improvement.

## Discussion

Model selection tools such as ModelTest [28] and its amino acid counterpart ProtTest [16] have been widely adopted for selecting the best fitting models for a given alignment. In this paper we show that, rather than simply selecting the best from a list of existing models, models of protein evolution can be tailored to specific alignments. Our NNMF framework has two primary strengths: 1) the model complexity adapts to fit the alignment, and 2) the dimensions along which the model can vary and the trajectory along which the complexity increases have been learnt, at least approximately, from a large collection of real alignments.

Since NNMF finds higher quality exchangeability matrices, we should expect it to benefit any application that uses such matrices. In this paper, we demonstrate an impact on phylogeny inference. Although we don't demonstrate it here, these rate matrices can also be used to construct scoring matrices for sequence alignments. A procedure for doing this, along with software for generating the scoring matrices, is outlined in [13]. Given that an alignment is required before NNMF can be used, an iterative procedure, in which a guide alignment obtained from a standard scoring matrix is used to estimate an NNMF model, would have to be adopted. A scoring matrix based on this model can then be generated to refine the alignment.

### Using more basis matrices

On our test alignments, we explored up to 40 basis matrices. This choice was motivated by computational considerations. The histogram of the optimal number of basis matrices for each dataset (Figure 7) suggests that using more basis matrices could lead to further improvement on some alignments. We provide basis matrices for the first 100 factorizations, so users can explore as many dimensions as their computational restrictions allow. It is worth pointing out that, when the number of basis matrices becomes 190, the NNMF model is equivalent to the REV model. This justifies the interpretation of the procedure as interpolating between a model with no flexibility and a fully flexible one.

### Other approaches

CodonTest [26] is a recently proposed approach to solving a similar problem using a different approach, but at the codon rather than amino acid level. A genetic algorithm is used to find an optimal number of non-synonymous rate classes, as well as an assignment of particular non-synonymous substitution rates to these classes. The difference in the ‘level’ of modeling (codon *vs* protein) is superficial: applying our approach to codon models would be straightforward, though at some extra computational expense. The approach of CodonTest is different, in that it explores a much larger space of possible parameter clusters. While the difference in levels prevents direct comparison, we expect the NNMF approach to gain some additional leverage over that of CodonTest, because the set of subspaces it explores is learnt from a collection of training alignments, while CodonTest does not incorporate this prior information.

During the final preparation of this manuscript we became aware of recent work by Zoller and Schneider [29] in which a similar problem is tackled using an approach based on dimensionality reduction, again in the context of codon models rather than amino acid models. They used principal components analysis (PCA) to estimate a set of basis matrices, and, as in our approach, constructed their final model as a linear combination of these basis matrices. PCA has the advantage of being more computationally efficient than NNMF, but it lacks the non-negativity constraints. It is thus possible that certain linear combinations of PCA basis matrices will yield rates that are smaller than 0. Zoller and Schneider [29] circumvent this problem by explicitly resetting all negative rates to 0. That their model is applied to codon level data prevents a direct comparison, but future work will surely necessitate comparing different methods of dimensionality reduction for this task. We see their work as an encouraging sign that there is fertile ground for applying dimensionality reduction to phylogenetic models of evolution.

### Practical recommendations

Our NNMF approach can be applied whenever a numeric model of amino acid evolution is required. The following procedure would appear sensible: First, estimate a guide tree using a fixed protein model. Then use the NNMF HBL program to find the best NNMF model. At this point, the model could be used to re-estimate the guide tree and iterate the NNMF procedure. Since each iteration should improve the model selection criterion (which is also bounded), this procedure should converge. Finally, the output can be converted to the form appropriate for the remaining analysis (phylogeny estimation, alignment etc). Some publicly available empirical rate matrices are provided with a fixed set of equilibrium frequencies. Importantly, our NNMF procedure used the empirical amino acid frequencies, and there are no such frequencies associated with any of our rate matrices, so any applications requiring equilibrium frequencies should use either the empirical frequencies, or estimate the equilibrium frequencies by maximum likelihood.

Rate variation may be introduced at any step. To save computation, one could use the NNMF HBL script without rate variation to obtain a rate matrix, and subsequently introduce rate variation. With more computational resources, rate variation can be included while optimizing over the combination weights. It is an open question whether including rate variation when estimating the original REV models (before the NNMF step) would significantly improve subsequent steps that also include rate variation. Results reported in [26] suggest that rate variation should be mostly orthogonal to estimating the relative substitution rates.

### An approximate solution to a harder problem

Learning basis matrices by NNMF can be seen as an approximation to a more computationally challenging problem. It is possible to express the likelihood function for the factorization directly:
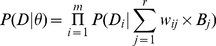
(4)where 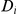 is the 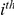 alignment in the training set, the likelihood within the sum is computed, as usual, using Felsenstein's pruning algorithm [4], and 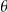 is the full collection of parameters, including weights and basis matrices. In this formulation, the rates in the basis matrices 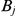 and the combination weights 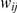 could all be optimized numerically to maximize the overall likelihood on the training data. However, obtaining this optimal solution would be computationally challenging – our NNMF procedure approximates this by finding separate REV models that maximize the likelihood on each alignment, and then finding the factorization that most closely recovers these REV models in the mean square error sense. The implicit assumption is that this factorization will also yield good likelihoods. The computational saving relative to the full solution occurs in part because the REV models can be optimized separately for each training alignment.

### Future avenues for research

Estimating a model of evolution that is specific to a single alignment clearly improves on the generalist approach. It is still, however, an incredibly coarse approximation to reality. The constraints and selective pressures on each site are most likely unique, but estimating a model for each site would be intractable, both computationally and statistically. Goldman *et al.* [30] took early steps in this direction, allowing the model of evolution to vary from site to site by using a Hidden Markov Model to capture the correlational structure across sites. Lartillot and Philippe [31] introduce a model that allows each site to belong to one of a number of classes, which differ in their equilibrium frequencies. A Dirichlet process prior is adopted to accommodate uncertainty about the number of classes, as well as the assignment of sites to classes. Le and Gascuel [32] also allow the substitution matrices to vary across sites. In their approach, they assume a small number (2 or 3) of distinct substitution processes, and their model treats each site as a random draw from one of these processes. This works well when clues about which process belongs to which site are available, but when the whole procedure is unsupervised the optimization appears to be difficult and sensitive to initial conditions [32], [33]. Developing unsupervised approaches for estimating such models with larger numbers of distinct processes is an intriguing avenue for future research.
